# Editorial: Hammer or telescope? Challenges and opportunities of science-oriented AI in legal and sociolegal research

**DOI:** 10.3389/frai.2024.1333219

**Published:** 2024-04-26

**Authors:** Nicola Lettieri, Alessandro Pluchino

**Affiliations:** ^1^National Institute for Public Policy Analysis, Rome, Italy; ^2^Department of Law, Economics, Management and Quantitative Methods, University of Sannio, Benevento, Italy; ^3^Department of Physics and Astronomy “E. Majorana”, University of Catania, Catania, Italy; ^4^INFN Sezione di Catania, Catania, Italy

**Keywords:** science-oriented AI, complexity theory, artificial intelligence, legal science, computational legal studies

## 1 The hammer and the telescope: on the dual nature of AI

In a brief but insightful introduction to artificial intelligence published in 2018, the cognitive scientist Boden (2018) identifies two main aims for using AI. One is purely scientific and consists of “using AI concepts and models to help answer questions about human beings and other living things;” the other is essentially technological and turns into “using computers to get useful things done.” As a matter of fact, if, on the one hand, it has a crucial role in fundamental advancements of science, AI is also an essential tool to accomplish practical purposes. Machine learning's ability to make predictions and identify hidden patterns within vast floods of data has indeed spawned a wide range of technological applications that often overshadow the ability of AI to improve the understanding of the world we live in.

In such a scenario, while intelligent technologies are seeping into any aspect of our lives, it makes sense to question the way we use AI and the implications of our choices in this respect. To use a metaphor, it makes sense to ask whether we want to use it as a “hammer” or a “telescope.” This is also real in the legal world where, despite a longstanding research tradition concerning artificial intelligence applications, the question has not been clearly posed in these terms. Articles in this Research Topic confirm this trend (Dresp-Langley, Loddo et al., Kontiainen et al., Sobkowicz, McCarthy-Jones). As it can be easily deduced from an even cursory review of the contributions published in this Research Topic, scholars' attention is primarily focused on the practical applications of artificial intelligence taken into account mainly for its implications for individual and collective rights. Yet, the scenarios to consider are vast and heterogeneous.

### 1.1 AI as a hammer

In general terms, the list of scenarios where AI becomes a tool to tackle practical problems is long and spans from healthcare—where Intelligent algorithms help medical professionals diagnose diseases more accurately and efficiently—to cybersecurity—where machine learning is used to perform automated threat detection and response. In the context of e-commerce and streaming platforms, again, AI-based recommender systems analyze user behavior, preferences, and historical data to provide personalized recommendations, helping explore new products, movies, or music. Finally, machine learning financial tools can help with fraud detection, risk assessment and algorithmic trading, identifying patterns of fraudulent activities, assessing creditworthiness and making data-driven investment decisions.

### 1.2 AI as a telescope

AI can also be used as a telescope, a scientific tool for exploration and discovery capable of expanding our understanding of reality by uncovering hidden relationships and providing new perspectives on complex phenomena. Such a function can be performed with different levels of intensity ranging from purely scientific (strong) uses of AI, to cases in which scientific and applicative objectives merge in the same activity (weak). An example of the first category occurs when machine learning is used in climate change research to analyze complex climate models, satellite data, and historical records to help identify trends, predict future scenarios, and provide insights into the impact of human activities on the environment. A “weak version” of the use of AI as a telescope could be found in AI-powered language models, like ChatGPT, if used to provide information, answer questions and assist researchers in various fields.

## 2 Legal hammers, legal telescopes

The hammer metaphor can also be brought into the legal domain, where AI could serve as a tool to streamline legal processes and enhance efficiency, acting as a source of valuable insights that can inform decision-making and strategies. As witnessed by a growing number of applications, machine learning algorithms can efficiently review and analyze large volumes of legal documents, contracts, and case law to extract relevant information, identify patterns, or flag potential issues. In performing information retrieval tasks, AI-powered tools can help practitioners by providing quick access to vast legal databases, suggesting relevant case law or statutes, and helping to draft legal documents. In contract analysis and management, the same tools can review contracts, extract key terms and clauses, identify potential risks or discrepancies, and assist in contract management processes. In document drafting, AI can automate the generation of standard legal documents, such as wills, non-disclosure agreements or employment contracts, saving time and reducing the risk of errors.

As to the “telescopic” use of AI, the “weak” version is probably the most widespread in the legal domain. Artificial intelligence allows us to analyze historical legal data and make predictions about the outcome of legal cases, helping lawyers to assess the potential risks and to evaluate strategies accordingly. AI algorithms can identify emerging legal trends, track regulation changes, or highlight significant court decisions, enabling legal professionals to stay informed and adapt their strategies. AI-driven chatbots can provide basic legal information, guide individuals through legal processes, or offer preliminary legal advice, increasing access to justice for more people. AI algorithms can also analyze public sentiment or social media conversations related to a legal case, providing insights into public opinion and potential impacts on the case. Again, AI-powered robotic judges pave the way to new forms of support for human judges. The automated analysis of vast amounts of legal data (precedents, statutes, and legal documents) can turn into solutions enhancing the efficiency and impartiality of legal decisions with positive spillovers in terms of reduction of human biases, certainty, equity and, finally, coherence of the whole legal system.

Despite the many reasons of interest for the “weak” forms of legal use of AI, the most challenging (and less explored) perspective appears to be the “strong” one. Recent developments in sociophysics, computational social science and complex networks theory clearly show that AI can provide a conceptual vocabulary and tools to improve the understanding of phenomena that play a crucial role in shaping the legal universe. Neural networks, agent-based simulations, evolutionary computing, and multilayer and higher-order interaction networks can shed indeed a new light on many of the building blocks underlying the complexity of law seen both as a normative architecture and a dynamic, emerging social construction.

Definitely, we can state that both interpretations of AI have merit as the way we conceive AI can legitimately shape our approach to its development and application. The perspective we can decide to adopt will obviously depend on the context, the objectives, and the ethical considerations surrounding the use of AI in a particular domain. Ultimately, the impact of these new technologies on society depends on the decisions and actions taken by individuals, organizations and policymakers. It is essential in any case to approach the development and deployment of AI with a thoughtful mindset, considering its potential benefits and risks to ensure it serves the collective wellbeing.

## Author contributions

NL: Conceptualization, Writing – original draft. AP: Conceptualization, Writing – original draft.
